# Puerperal Eclampsia, Treated by Subcutaneous Injections of Morphia

**Published:** 1860-09

**Authors:** 

**Affiliations:** Strasburg. (Bull. de Therap.)


*Puerperal Eclampsia, treated by Subcutaneous Injections of Morphia.* By Scanzoni, of Strasburg. (Bull. de Therap.)

The attention of the public has recently been awakened by the statements of M. Wood, and afterwards of Messrs. Hunter and Bahier, with regard to the beneficial effects of subcutaneous injections, and particularly of narcotic injections. I could easily swell the list of successful cases of neuralgia and hyper-æsthesia healed by this plan; but I prefer to relate a case of Eclampsia which sustains the conclusions of Hunter, by showing that we possess in these subcutaneous injection sa means of acting upon and controlling irritation of the brain more rapid and more cer. tain than by the administration of the same agents by the stomach.

It will not be disputed that opium and its preparations occupy the first place in the treatment of puerperal eclampsia. For myself, I have been convinced by observation that a species of intoxication from opium conduces more surely to a favorable termination in this disease than the use of all other means recommended against this cruel malady. Unfortunately the administration of opium or morphia is not always an easy task. Coma, or the rapidity with which spasms succeed each other, oppose the administration of the remedy by the mouth, and enemata are immediately rejected. A mode of overcoming these difficulties is offered by the hypo-dermic treatment. Numerous experiments with this mode of treatment have satisfied me that if its effects cannot be always said to be permanent, as in the *cure* of neuralgias, it nevertheless produces in a very short space of time (in some cases a few minutes suffice) effects which leave no question as to the action of the opium on the brain. The contents of a single syringe half filled with meconate of morphia, and containing about 2½ grammes (125 milligrammes of opium) thrown into the cellular tissue, constantly produce heaviness, drowsiness, headache, pain at the præcordia, a sense of contraction at the œsophagus, and even vomiting, with functional depression of the nerves of the breast (*sein*), and these phenomena may progress, if the dose be stronger, even to somnolence. Considered in connection with facts already known as to the use of the hypodermic treatment in *delirium tremens*, mania, chorea, tetanus, &c., these results induced me to apply this method to the treatment of puerperal eclampsia with the most gratifying results; for, as will be seen in the following case, this plan reduced the number of attacks from three in one hour and three-quarters to two in nine hours. This diminution in the frequency of attacks is the more remarkable since experience has shown that the paroxysms not only become more .and more intense in proportion to the advance in the labor, but that as a general rule the interval between them is also shortened. I am far from believing the hypodermic use of morphia to be an infallible panacea against this fearful disease, but it strikes me that the character of the case is such as should engage medical men to try this plan.

*Case.*—D***, æt. 21, a strong and robust primipara, was brought into the labor ward at three quarters past seven on the morning of the 8th June, 1859. Labor had commenced during the night, upon which she was seized with violent nervous attacks, during which she lost consciousness. No further information was given as to character or duration of these attacks. The patient remembered nothing which had happened during the night. The whole body, and particularly the legs, were œdematus. The tongue bore on its right side marks of numerous bruises from the teeth, the fundus uteri was at the epigastrium, and its consistence firm. "The touch" being instituted, the vaginal portion of the neck of the uterus was found effaced, the orifice dilated to the size of a piece of fifty centimes, the waters formed with presentation of the head. The urine was very albuminous, and offered under the microscope many fibrinous cylinder casts to view. With this knowledge came the probability that the attacks of the preceding night were eclamptic, and this presumption became a certainty at 8 o'clock, at which time she was seized by a second well marked attack of eclampsia, which lasted some minutes, after which time her senses returned, and she answered questions, though slowly. A third attack came on at 8.45, a fourth at 9.45, a fifth at 11.45, and a sixth, which was the strongest of all, at 5 o'clock. Consciousness was lost after the fourth attack, nor did it return, and the respiration became stertorous. At 10 o'clock v. s. ad 250 grm. enemata with 25 drops of an opiate; bath for the whole body, and cold irrigations of the head while the body was in the bath. As opium could not be introduced into "the natural ways," a solution of meconate of morphia was injected under the skin at three different times; the first with 25 centigr.; the whole being 75 centigrammes of opium. The labor progressed slowly, the pain being very rare. At 3 o'clock at night the waters broke, the orifice was the size of a three-franc piece, the head high above the superior strait, and the heart-sounds still very distinct. From this time dilatation was more rapid. At 7 the os was as large as a piece of six livres, extensible and dilatable, but the head still high and immovable. Insensibility complete, with profound coma. In this dangerous condition I dreaded, notwithstanding the position of the head and the incomplete dilatation of the os, to put on the forceps. This of course was not very easy, but the extraction presented no difficulty. After a few tractions we extricated a child which breathed feebly, but did not fail shortly after to scream lustily. The afterbirth followed. There was no attack during the delivery. Wine with ten drops of tincture of amber and musk were given hourly, with the effect of rousing her a little, but without awakening consciousness. At 11 o'clock there was a short and feeble paroxysm, after which she became agitated and tried to escape; towards morning she became more calm. At 9 A. M. she answered when spoken to loudly; during the whole day she remained as if intoxicated. Pulse 128. The musk was discontinued and she was ordered lemonade. Towards evening the belly became a little painful. During the night there were several slight attacks of mania, during which she constantly attempted flight. In the morning she answered questions rationally. Pulse 108. The œdema has diminished, belly painful; there are numerous fine and coarse rales in either lung, with difficult respiration. Warm bath, lemonade, infus. ipecacuanha, with oxymel scillæ and syrup of diacodium. In the evening the patient had fully recovered her intelligence. Pulse 132.

June 11: Passed a quiet night; a table-spoonful of ol. ric. was followed by several large liquid stools. Œdema of the labia majora—belly still painful. Same potion as yesterday with 1.5 grain opium. Cataplasms to the abdomen and fomentations of the genitals with chamomile.

June 12: Quiet night—tranquil sleep. Expectoration free; rales still present. Pulse 120. Warm bath. Omit opium in the potion. Urine slightly albuminous but no cylinders.

June 13: Patient's state good; no œdema; belly indolent. During the night there is involuntary escape of urine which yields to the continued presence of a sound. All medication is suspended, and the patient put on good diet, with a glass of chalybeate water every morning. The 17th, albumen was no longer found, and on the 12th July, the patient left the hospital with her child, with advice to continue the use of the chalybeate for a considerable time.—*Md. & Va. Med. Jour.* McK.

